# Twin–Twin Transfusion Syndrome: study protocol for developing, disseminating, and implementing a core outcome set

**DOI:** 10.1186/s13063-017-2042-0

**Published:** 2017-07-14

**Authors:** Asma Khalil, Helen Perry, James Duffy, Keith Reed, Ahmet Baschat, Jan Deprest, Kurt Hecher, Liesbeth Lewi, Enrico Lopriore, Dick Oepkes, On behalf of the International Collaboration to Harmonise Outcomes for Twin–Twin Transfusion Syndrome (CHOOSE)

**Affiliations:** 1 St George’s University of London, Blackshow Road, Tooting, London, SW17 0QT UK; 2 Nuffield Department of Primary Care Health Sciences, University of Oxford, Oxford, OX2 6GG UK; 3 Twin and Multiple Births Association (TAMBA), The Manor House, Manor Park, Church Hill, Aldershot, GU12 4JU UK; 4 The Johns Hopkins Center for Fetal Therapy, 600 North Wolfe, Nelson 228, Baltimore, MD 21287 USA; 5 Department of Obstetrics and Gynecology, University Hospitals of KU Leuven, Herestraat 49, 3000 Leuven, Belgium; 6 Department of Obstetrics and Fetal Medicine, University Medical Center Hamburg-Eppendorf, Neues Klinikum, Gebäude O10 Martinistraße 52, 20246 Hamburg, Germany; 7 Department of Pediatrics, Leiden University Medical Center, K-06-35, P.O. Box 9600, 2300RC Leiden, The Netherlands; 8 Division of Fetal Medicine, Department of Obstetrics, Leiden University Medical Center, K-06-35, P.O. Box 9600, 2300RC Leiden, The Netherlands

**Keywords:** Twin–Twin Transfusion Syndrome, Core outcome set, Modified Delphi method

## Abstract

**Background:**

Twin–Twin Transfusion Syndrome (TTTS) is associated with an increased risk of perinatal mortality and morbidity. Several treatment interventions have been described for TTTS, including fetoscopic laser surgery, amnioreduction, septostomy, expectant management, and pregnancy termination. Over the last decade, fetoscopic laser surgery has become the primary treatment. The literature to date reports on many different outcomes, making it difficult to compare results or combine data from individual studies, limiting the value of research to guide clinical practice. With the advent and ongoing development of new therapeutic techniques, this is more important than ever. The development and use of a core outcome set has been proposed to address these issues, prioritising outcomes important to the key stakeholders, including patients. We aim to produce, disseminate, and implement a core outcome set for TTTS.

**Methods:**

An international steering group has been established to oversee the development of this core outcome set. This group includes healthcare professionals, researchers and patients. A systematic review is planned to identify previously reported outcomes following treatment for TTTS. Following completion, the identified outcomes will be evaluated by stakeholders using an international, multi-perspective online modified Delphi method to build consensus on core outcomes. This method encourages the participants towards consensus ‘core’ outcomes. All key stakeholders will be invited to participate. The steering group will then hold a consensus meeting to discuss results and form a core outcome set to be introduced and measured. Once core outcomes have been agreed, the next step will be to determine how they should be measured, disseminated, and implemented within an international context.

**Discussion:**

The development, dissemination, and implementation of a core outcome set in TTTS will enable its use in future clinical trials, systematic reviews and clinical practice guidelines. This is likely to advance the quality of research studies and their effective use in order to guide clinical practice and improve patient care, maternal, short-term perinatal outcomes and long-term neurodevelopmental outcomes.

**Trial registration:**

Core Outcome Measures in Effectiveness Trials (COMET), 921 Registered on July 2016.

International Prospective Register of Systematic Reviews (PROSPERO), CRD42016043999. Registered on 2 August 2016.

## Background

Twin–Twin Transfusion Syndrome (TTTS) is a serious pathology exclusive to monochorionic twin pregnancies whereby unbalanced transfusion across the placental vascular anastomoses leads to volume of red cell imbalance between the twins [1]. The severity of TTTS is most commonly classified using the Quintero staging system [1]. In stage 1, the optimal treatment approach (conservative versus intervention) remains under debate. In more advanced stages, intervention is usually recommended to increase the chance of survival of at least one twin. In severe TTTS the mortality rate is as high as 90% if untreated [2, 3]. Even with treatment, TTTS is associated with an increased risk of perinatal morbidity compared with uncomplicated monochorionic pregnancies, with neurological and cardiac complications reported, as well as a significant risk of preterm birth [2–8].

Several treatment interventions have been described for TTTS: fetoscopic laser surgery, amnioreduction, septostomy, expectant management, and termination of pregnancy. Over the last decade, fetoscopic laser surgery has become the mainstay of treatment [9]. Several studies have compared the different techniques and appear to show that fetoscopic laser surgery is better than amnioreduction in terms of twin survival and neurodevelopmental morbidity [10–13]. More recently, comparison of the different techniques of fetoscopic laser ablation has been made, with the Solomon technique showing improved outcomes compared with selective ablation [14, 15]. Given the high potential for morbidity and mortality in TTTS, there is a need for robust guidance on the safest course of management. With the advent and ongoing development of new therapeutic techniques, this is more important than ever. The literature to date reports on many different outcomes, making it difficult to compare results or combine data from individual studies, limiting the potential of research to guide clinical practice.

Core outcome sets are agreed, clearly defined minimum sets of outcomes that can be measured in a standardised manner and reported consistently [16]. Acknowledging that inconsistencies in outcome reporting can be disruptive to progress in our speciality, 78 editors of journals of women’s health came together to form a consortium to support the development, dissemination, and implementation of core outcome sets [17].

Recently, a core outcome set was developed for the evaluation of interventions to prevent preterm birth, involving 228 participants from five stakeholder groups. The stakeholders were from 27 countries. Using the Delphi method, a core outcome set to be used in future trials was constructed [18].

**Fig. 1:**
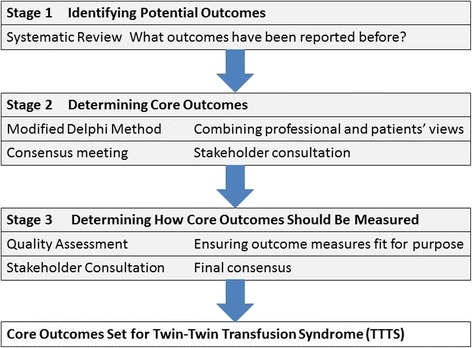
Stages of developing a core outcome set for Twin–Twin Transfusion Syndrome

Our objective is to produce, disseminate, and implement a core outcome set for TTTS following the steps outlined in Fig. 1.

## Methods

### Prospective registration

This study has been registered with the International Prospective Register of Systematic Reviews (PROSPERO) (registration number: CRD42016043999) and The Core Outcome Measures in Effectiveness Trials (COMET) initiative (registration number: 921). We will follow guidance set out by the PRISMA Statement for Reporting Systematic Reviews and Meta-Analyses of Studies that Evaluate Health Care Interventions [19] for this systematic review.

### Ethical review

The National Research Ethics Service has advised that ethical approval is not required for the Delphi survey because it is considered a service evaluation.

### Steering group

An international steering group, including healthcare professionals, researchers, and patient representatives, has been formed to guide the development of this core outcome set. The steering group was approached and invited based on their expertise as clinicians and researchers in TTTS. The steering group has been established to make decisions regarding the study’s methods, for example determining the scope of the core outcome set and selecting appropriate consensus methods. Whilst the steering group will oversee the process, further stakeholders will be involved in the consensus-forming process and anyone, anywhere is welcome to suggest an outcome to be entered into the consensus process and participate in the prioritisation of outcomes.

### Scope of the core outcome set

This core outcome set will apply to all therapeutic interventions for TTTS. The set will not be limited by the type of intervention, the setting in which it is administered, or the gestation at which it is provided. TTTS will be defined as a monochorionic twin or triplet pregnancy with oligohydramnios in one amniotic sac and polyhydramnios in the other [1]. We are not seeking to reach consensus on the standardisation of other aspects of study design or the definition or staging of TTTS.

### Identifying potential core outcomes

We will conduct a systematic review to identify what outcomes have been reported previously. We will search the Cochrane Central Register of Controlled Trials (CENTRAL) as well as EMBASE and MEDLINE from inception to August 2016 to identify all trials and observational studies reporting outcomes following intervention for TTTS using defined MeSH descriptor terms (‘twin twin transfusion syndrome’; ‘TTTS’; ‘interventions’). We will include all randomised controlled trials and observational studies that report an outcome following any intervention for TTTS. We will exclude case reports, case series (*n* < 3), editorials, and review articles. No data or language limits will be applied. All studies identified in the search will be screened from the title and abstract. The full-text article will be reviewed for all studies meeting the inclusion criteria and those where this cannot be decided from the abstract alone. Identified studies will be reviewed by two reviewers and any discrepancies resolved by discussion with a third reviewer. The studies will be assessed using a pre-defined proforma to gather the following information: year of study, journal, journal impact factor, study design, sample size, intervention undertaken, primary and secondary outcomes, outcome measure instruments, and funding source. The quality of outcome reporting will be assessed using the six-point Management of Otitis Media with Effusion in Cleft Palate scoring system which asks the following: was a primary outcome stated (one point); was the primary outcome clearly defined for reproducible measures (one point); were the secondary outcomes clearly stated (one point); were the secondary outcomes clearly defined for reproducible measures (one point); do the authors explain the choice of outcome (one point); and are the methods used designed to enhance quality of measures appropriate (one point) [20]? The systematic review will be reported following the PRISMA statement criteria [19]. All identified outcomes will be entered into an outcome inventory and organised into the following categories: survival outcomes, fetal outcomes, short-term neonatal outcomes, long-term neonatal outcomes, obstetric outcomes, and surgical/operator outcomes. These outcomes will be reviewed and discussed by the steering group with particular emphasis on reducing duplication of outcomes caused by varying terminologies and grouping very similar outcomes together in order to make the final outcome inventory clear and succinct. Following agreement, the inventory will be entered into the modified Delphi method. The wording of the outcomes will be decided in collaboration with the patient representatives (Twin and Multiple Births Association (TAMBA)).

### Determining core outcomes

The core outcomes will be determined using a modified Delphi method. The Delphi method is a long-established tool for achieving a convergence of opinion on a particular subject by gathering data from respondents with expert knowledge of that particular subject. It allows consensus-building by using a series of questionnaires to extract opinion from participants. Web-based Delphi tools facilitate international data collection and are largely considered acceptable to the user [21, 22].

All categories of stakeholder, including health professionals, researchers, and people with lived experience of or expertise in TTTS, will be invited to take part. The recruitment strategy has been designed to ensure that people with assorted experiences of TTTS from diverse demographic backgrounds and geographical locations can be recruited. Recruitment will be facilitated by national and international patient organisations, including TAMBA, and national and international professional organisations, including the Royal College of Obstetricians and Gynaecologists (RCOG), International Society of Ultrasound in Obstetrics and Gynaecology (ISUOG), and International Society of Twin Studies (ISTS), advertising the study within their newsletters, online forums, and social media feeds. Potential participants will be able to register their interest online and will be sent Delphi survey instructions written in plain language. When participants register to complete the survey, they will complete a questionnaire recording demographic details, for example age, ethnic group, and country, and information pertaining to their experiences or expertise of TTTS. Participants who fail to complete the Delphi survey will be asked about their reasons for dropping out. We will aim to recruit 18 participants from each stakeholder group. If this is not achieved, the steering committee will review the invitation process and re-advertise prior to commencing the Delphi process. The Delphi method will follow the following steps.

### Round one

All participants will be invited to register with the online survey and will be allocated a unique identifier to enable anonymisation of their responses. They will be asked to score individual outcomes on a nine-point Likert scale anchored between 1 (labelled ‘of limited importance for making a decision’) and 9 (labelled ‘critical for making a decision’). This scale was devised by the Grading of Recommendations Assessment, Development and Evaluation (GRADE) working group to facilitate the ranking of outcomes according to their importance and has been adopted widely by core outcome set developers [23]. There will also be an opportunity for participants to suggest new outcomes in a free text box and these will be considered by the steering group for incorporation into the second survey round. The survey will remain open for 6 weeks with a weekly email reminder for invited stakeholders to complete the survey.

### Round two

Responses from the first round will be disseminated back anonymously to participants before the second survey round commences. They will be split into individual response, stakeholder group response, and total group response. All outcomes will be carried forwards into the second round and any additional outcomes suggested by participants will be included. The mean value for the total group response will be visible for each outcome in the second round of the survey and participants will be asked to re-score the outcome with this new information. This repeated reflection and re-scoring promotes convergence of opinion to form consensus upon core outcomes [21]. A standardised definition will be applied to this round’s results, enabling core outcomes to be identified:

- consensus in (classify as a core outcome)—more than 70% of participants in each stakeholder group score outcome ‘critical for decision making’ (score 7–9) and less than 15% of participants in each stakeholder group score outcome ‘of limited importance for decision making’ (score 1–3);
- consensus out (do not classify as a core outcome)—more than 70% of participants in each stakeholder group score outcome domain ‘of limited importance for decision making’ (score 1–3) and less than 15% of participants in each stakeholder group score outcome domain ‘critical for decision making’ (score 7–9); or
- no consensus (do not classify as a core outcome)—anything else.

These cut-off values have been used successfully in the development of core outcome sets previously, including in a similar population with a similar stakeholder population [18, 20]. Defining consensus a priori will reduce bias that could be incurred with a post-hoc definition to fit the beliefs of the research team. Results will be analysed using SPSS 24.0 and displayed as median with interquartile range in frequency tables and as box plots. The results will be reviewed by the steering group to decide whether a further round is indicated due to a lack of consensus being formed.

### Stakeholder meeting

The results of the Delphi survey will be discussed in a consensus meeting where the main objective will be to address outcomes not reaching consensus after the Delphi method and approve a final core outcome set for interventions in TTTS. To ensure unbiased consensus formation amongst a group of varied participants, the steering committee will ensure that the meeting is informal, inclusive, participatory, and values all opinions. For those outcomes where consensus has not been agreed by the Delphi method, a final anonymous vote will be made at the meeting. This will be facilitated using smartphone/touchpad technology, allowing all present to vote anonymously at the same time. Scoring will be done using the Likert score with the aforementioned definition of consensus remaining the same. Outcomes that do not reach consensus at this stage will be rejected and the final core outcome set agreed.

### Failure to agree consensus

We have chosen the Delphi method as a consensus technique because it offers the advantage of converging large numbers of opinions without the need for participants to be together and it allows individuals to decide on their own without being influenced by others in a group setting. It is also of relatively low cost. However, the method does have some limitations: it depends highly on participant motivation to put thought and effort into each answer and to see the process through all of the rounds. The Delphi method is also fallible where there are large differences in opinion, and may lead to these not being investigated fully and, in turn, relevant outcomes being rejected unnecessarily [24]. As described earlier, if there are some outcomes that do not meet consensus, a vote will be cast at the stakeholder meeting. If it becomes apparent early in the process that the Delphi method is not working in this scenario, the steering group will re-evaluate the design of the process and consider other methods.

### Measuring core outcomes

Once the core outcome set has been agreed and established it will be necessary to determine how the variables should be measured. The steering group will consider all tools for this process including expert opinion as well as the ongoing work of The Core Outcome Measurement Instrument Selection (COMIS) project, which aims to develop validated tools for measuring outcomes [25]. This project has developed guidelines for the selection of outcome measurement instruments by those developing core outcome sets, including the identification of existing outcome measure instruments through a literature search or systematic review. We will therefore collect details of outcome measurement instruments as well as outcomes in our systematic review. Once we have analysed the range and heterogeneity of outcome measure instruments we will be better placed to decide how to proceed further with agreeing on set measures. The COMIS project emphasises the importance of the feasibility of any measures agreed and this is an important consideration for our international stakeholders who may not all have access to the same resources. We will aim to outline high-quality outcome measures for each core outcome and the study will not advocate the use of a single outcome measure if several high-quality outcome measures are identified for a single outcome. If no high-quality outcome measures exist, this will be acknowledged.

### Dissemination and implementation

The steering group will aim to disseminate the core outcome set as widely and effectively as possible. We will aim to describe the core outcome set through publication in a relevant journal as well as presenting to our peers at meetings. The Core Outcomes in Women’s and Newborn’s Health (CROWN) initiative is recognised by RCOG and the Royal Australian and New Zealand College of Obstetricians and Gynaecologists (RANZCOG), and we would hope they would endorse the development of this core outcome set and assist in disseminating our findings to clinicians worldwide. TAMBA will use their publicity channels to further share the core outcome set with both healthcare professionals and patients. With the CROWN initiative now supported by 78 journals, researchers will have more obligation to engage with the core outcome set when it comes to planning their studies.

## Discussion

The development and implementation of core outcome sets is likely to be very beneficial to the design and reporting of clinical studies, systematic reviews, and clinical guidelines. This should ultimately improve clinical care and patients’ experience. The importance of such an initiative has been acknowledged by a number of key national and international organisations.

### Improving the selection of the outcome of clinical studies

The Standard Protocol Items: Recommendations for Interventional Trials (SPIRIT) statement supported by funders of health research, such as the National Institute of Health Research (NIHR), recommends the use of core outcome sets. The use of standard core outcome sets would enhance comparability of clinical trials and facilitate the conduct of prospective meta-analyses using individual patient data.

### Facilitating the evidence synthesis and reporting of clinical studies

The CROWN initiative, supported by 78 speciality journals, including the Cochrane Pregnancy and Childbirth Group, has resolved to implement core outcome sets. These journals would expect authors to report the study results for the core outcomes and draw their conclusions based on these outcomes rather than non-core or surrogate outcomes.

### Enhancing the ability to develop robust clinical guideline

The National Institute for Health and Care Excellence (NICE) supports the use of core outcome sets during evidence scoping and synthesis [17, 26]. The NICE methodology for assessment of the quality of the evidence takes into account whether the data of interest were reported as a core, non-core, or surrogate outcome. This initiative to improve the quality and consistency of outcomes investigated and reported by researchers can in turn lead to the development of guidelines based on clearer and stronger evidence to help all clinicians offer the best interventions for their patients.

### Developing a network which can support an international collaboration

The team of involved key stakeholders has the potential to set up an international network, which could be a potent vehicle for the development of international guidelines, registries, and setting research priorities for TTTS. In the context of TTTS, this could potentially have a profound impact on morbidity and mortality rates in the long term.

## Trial status

At the time of manuscript submission, the systematic review process has commenced and strategic planning for the Delphi method consensus-building exercise is underway.

## Abbreviations

CENTRAL: Cochrane Central Register of Controlled Trials
COMET: Core Outcome Measures in Effectiveness Trials Initiative
COMIS: Core Outcome Measurement Instrument Selection
CROWN: Core Outcomes in Women’s and Newborn’s Health
GRADE: Grading of Recommendations Assessment, Development and Evaluation
ISTS: International Society of Twin Studies
ISUOG: International Society of Ultrasound in Obstetrics and Gynaecology
MeSH: Medical Subject Headings
NICE: National Institute for Health and Care Excellence
NIHR: National Institute of Health Research
NRES: National Research and Ethics Service
PRISMA: Preferred Reporting Items for Systematic Reviews and Meta-analyses
PROSPERO: International Prospective Register of Systematic Reviews
RANZCOG: Royal Australian and New Zealand College of Obstetricians and Gynaecologists
RCOG: Royal College of Obstetricians and Gynaecologists
SPIRIT: Standard Protocol Items: Recommendations for Interventional Trials
TAMBA: Twin and Multiple Births Association
TTTS: Twin–Twin Transfusion Syndrome
